# Imaging of hepatocellular carcinoma and image guided therapies - how we do it

**DOI:** 10.1186/s40644-017-0110-z

**Published:** 2017-03-04

**Authors:** Jonathon Willatt, Julie A. Ruma, Shadi F. Azar, Nara L. Dasika, F. Syed

**Affiliations:** Veterans Administration, University of Michigan, Ann Arbor, MI USA

## Abstract

Treatment options for hepatocellular carcinoma have evolved over recent years. Interventional radiologists and surgeons can offer curative treatments for early stage tumours, and locoregional therapies can be provided resulting in longer survival times. Early diagnosis with screening ultrasound is the key. CT and MRI are used to characterize lesions and determine the extent of tumour burden. Imaging techniques are discussed in this article as the correct imaging protocols are essential to optimise successful detection and characterisation. After treatment it is important to establish regular imaging follow up with CT or MRI as local residual disease can be easily treated, and recurrence elsewhere in the liver is common.

## Background

Hepatocellular carcinoma (HCC) is the most common liver cancer and the fifth most common cancer worldwide. It results in between 250,000 and 1 million deaths globally per annum [1]. The number of deaths per year in HCC is close to that of the incidence throughout the world, which emphasizes the high case fatality rate of this aggressive cancer [1].

80% of HCC cases are associated with chronic hepatitis B and C virus infections [2]. Alcoholic liver disease is a risk factor in younger age groups, and the combination of alcoholic liver disease and viral hepatitis substantially increases the risk for the development of cirrhosis and HCC. The obesity epidemic has resulted in a growing population of patients with non-alcoholic fatty liver disease, cirrhosis and HCC [3].

In the United States, HCC, with its link to the hepatitis C epidemic, represents the fastest growing cause of cancer mortality overall and the second fastest growing cause of cancer deaths among women [4].

## Surveillance

The AASLD (American Association for the Study of Liver Diseases) recommends screening for the following high-risk groups: Asian male hepatitis B carriers over age 40, Asian female hepatitis B carriers over age 50, hepatitis B carriers with a family history of HCC, Africans and African Americans with hepatitis B, cirrhotic hepatitis B carriers, individuals with hepatitis C cirrhosis, individuals with stage 4 primary biliary cirrhosis, individuals with genetic hemochromatosis and cirrhosis, individuals with alpha 1-antitypsin deficiency and cirrhosis, individuals with cirrhosis from other etiologies [5].

We scan patients with cirrhosis from any etiology every 6 months with ultrasound [5, 6]. Ultrasonography remains the primary imaging modality of choice for HCC surveillance. It is more cost-effective than CT and MRI, and more widely available. A meta-analysis reported a sensitivity of 94% in detecting lesions and a specificity of >90% [7], although the figures were less favourable for lesions measuring less than 2 cm. The sensitivity for early HCC is 63%. Although our liver clinic routinely uses alpha-fetoprotein as an adjunct to imaging screening, it is acknowledged that it is neither sensitive nor specific for early diagnosis of HCC [8].

Once a nodule is detected, further follow-up depends on the size of the lesion(s), with both the American Association of the Society of Liver Diseases (AASLD) and the European Association for the Study of the Liver, European Organisation for Research and Treatment of Cancer (EASL–EORTC) using a threshold for further management of 1 cm. For nodules measuring less than 1 cm, the patient returns for a repeat ultrasound at 3 or 4 months. For nodules greater than 1 cm, the patient undergoes a dynamic contrast enhanced computed tomography (CT) or magnetic resonance imaging (MRI). The diagnosis of HCC is then determined by imaging characteristics.

## CT or MRI

Unlike most other cancers, HCC can be diagnosed on imaging studies only without tissue sampling confirmation. Currently, all major consensus groups support the diagnosis of HCC with contrast-enhanced multiphasic CT, or with MRI using an extracellular contrast agent [5, 6]. Studies have shown a similar or slightly better diagnostic performance of dynamic MR imaging compared with multiphasic CT [9, 10] although the difference in sensitivities is small [11–13].

The decision to perform one over the other may depend on institutional preferences, individual patient needs, and availability. Advantages of CT over MRI include lower cost, increased availability, and faster scan times. Faster scan times in particular can be an advantage in the context of a cirrhotic population with multiple morbidities and difficulty in cooperating with the breath hold requirements of MRI. Advantages of MRI include the capacity to evaluate a greater variety of tissue properties including fat content, restriction of diffusion, or T2-weighted increased signal, all of which may help in lesion detection and characterization. Lack of ionizing radiation may also be a consideration in younger patients.

## Ultrasound technique

We use a standard diagnostic 3–5 Mhz linear curved array probe to evaluate the liver. Subcostal real time imaging is performed of the left lobe, followed by intercostal and subcostal views of the right lobe. Both transverse and longitudinal projections are performed. Ask the patient to adopt a left lateral decubitus position for visualization of the right lobe after initially imaging in the supine position.

Initially information on the echogenicity and coarseness of the liver echotexture is assessed, as well as smoothness or nodularity of the liver surface. Then we look for focal lesions. Comparison with prior studies is essential to assess for stability or change in small hypoechoic or hyperechoic nodules. Once a new nodule or a change in a nodule is identified the patient goes on to CT or MRI, often on the same day.

We look at the hepatic vasculature. Although we do not do a full Doppler evaluation of the liver, we always look at the portal vein for direction of flow with both colour and spectral techniques and for any filling defects suggestive of tumor or bland thrombus.

An interval increase in the degree of splenomegaly can indicate a worsening of portal hypertension, so we measure the spleen as a final component of the study (Table 1).

**Table 1:** Summary of imaging techniques for ultrasound, MRI and CT

Ultrasound liver	MRI liver	CT liver
3–5 MHz Curvilinear Probe Transverse and longitudinal imaging, to include supine and left lateral decubitus positions Doppler evaluation of the portal vein Spleen measurement	Sequences Cor T2-w Single Shot Fast Spin Echo +/−Fat Saturation (FS) Ax T2-w Fast Spin Echo FS Ax Diffusion Weighted Imaging Ax dual gradient echo Ax 3D Spoiled Gradient Echo FS pre and post dynamic contrast enhancement (and coronal reconstruction in venous phase) Ax 2D Spoiled Gradient Echo FS post contrast delayed phase 10 ml Gadavist Subtraction imaging provided	Non contrast phase IV Contrast: Iohexol 100 mL Bolus tracking for arterial phase (average 30 s) Venous phase 65 s Delayed phase 240 s Single breath for each phase Injection rate min 4 ml/s Slice thickness 3 mm no overlap Coronal reconstructions in venous phases provided. Subtraction imaging optional

## MRI technique

We perform MRI of the liver at 1.5-T field strength, although a 3.0-T field strength can also be used [14]. A phased-array coil is routinely employed. Our protocol for imaging the cirrhotic liver includes T1-weighted gradient-recalled echo (GRE) in-phase and opposed-phase sequences, a moderately T2-weighted FSE sequence with an echo time of 80–90 msec, diffusion weighted imaging (DWI) and multiphase T1-weighted dynamic gadolinium-enhanced sequences.

A heavily T2-weighted sequence (echo time, ≥120 msec) can help to distinguish between cystic and solid lesions and a fast sequence, such as single-shot FSE (or half-Fourier acquisition turbo spin-echo—half-Fourier rapid acquisition with relaxation enhancement), is used for this purpose.

The sequences used can vary according to vendor and personal preferences. To improve image quality, sequences should be performed during suspended respiration or should be respiratory averaged (some T2-weighted sequences). Suspending respiration at end expiration produces more consistent breath holding compared with end inspiration but is more difficult for patients [15]. GRE sequences are widely used for T1-weighted imaging. Using a dual gradient-echo sequence that allows simultaneous acquisition of the earliest opposed-phase and in-phase images minimizes misregistration and improves the characterization of focal lesions and diffuse liver disease [16]. The acquisition of the earliest opposed-phase echo (2.2 msec at 1.5-T and 1.15 msec at 3-T imaging) followed by the subsequent in-phase echo enables the distinction between signal intensity loss caused by the presence of lipid seen on opposed-phase images and signal intensity loss due to susceptibility artifact from hepatic iron deposition, which is exaggerated on the longer of the two echoes (usually in phase).

Three-dimensional gadolinium-enhanced GRE sequences are preferred to two-dimensional GRE sequences because of the thinner sections obtained, which improve lesion detection and permit multiplanar image reconstructions for presurgical planning [17]. Section thickness should not exceed 4 mm for three-dimensional sequences and 6 mm for two-dimensional sequences. Contrast agent bolus timing is strongly recommended, based on our experience and review of the literature [18], to ensure the consistent capturing of the arterial-dominant phase; fixed delay is not a reliable method in this patient population. Options include use of a test bolus and various automated detection methods [19]. Hypervascular HCC is most conspicuous in the late arterial phase and can be missed if the arterial-dominant phase images are acquired early [20]. A timing bolus is not essential if rapid multiphase arterial imaging is performed. To improve lesion characterization—for example, to detect washout or delayed contrast material retention of hemangioma and cholangiocarcinoma—multiphase dynamic gadolinium-enhanced imaging should include three contrast-enhanced phases or more. We routinely acquire four sets of images after gadolinium-based contrast material injection in the arterial-dominant (automated timing, usually 20–35 s), venous (60–90 s), interstitial (120–150 s), and delayed (5 min) phases of hepatic enhancement. The highest spatial resolution should be used without compromising signal intensity, taking into account patients’ breath-holding capacity. Parallel imaging techniques can be applied to improve spatial resolution and/or reduce acquisition time. However, these techniques should be implemented with care, because they can result in image artifacts and reduced lesion conspicuity [21].

We find ourselves frequently dependent on subtraction imaging because of the intrinsic high signal demonstrated by nodules in the cirrhotic liver, including regenerative, dysplastic and malignant nodules. Intrinsic high signal can also be demonstrated in successfully treated HCC [22]. Unenhanced images can be subtracted from arterial-phase gadolinium-enhanced images to assess for arterial enhancement in nodules [23]. Subtraction can be performed if the unenhanced and gadolinium-enhanced imaging sequences are identical, if the imager is not retuned between acquisitions, and if there are no image rescaling issues. Acquiring the unenhanced and gadolinium-enhanced images in a single series rather than in separate series minimizes these differences and is possible with most systems. Patients should be instructed to hold their breath in a similar fashion during all sequences to minimize misregistration artifacts, which appear as a bright line at the edge of organs owing to incomplete overlap. At this point the ability of the MR radiographer or technician to coach the patient is crucial. Consistent breath holds are important in many MR sequences because of the lengths of the scans, but for subtraction imaging it is impossible to overemphasise the absolute requirement for good breath holds. If the patient, despite careful coaching, is unable to hold his/her breath, then CT, despite the change in modality, may be the better form of imaging.

Diffusion weighted imaging increases the detection rate of HCC, particularly for small tumours [24–26]. B-values typically used include one in the low range (0–50 s/mm2) and one in the intermediate-to-high range (400–800 s/mm2). We find that the DWI sequence frequently helps us to lean in favour or against small arterial enhancing lesions with equivocal washout as HCC, as well as assisting us in bringing our attention to small lesions which are inconspicuous on contrast-enhanced sequences [27]. Tumors can be obscured on DWI because of the increased DWI signal in fibrotic liver parenchyma and subsequent decreased lesion to liver contrast [28]. In addition, DWI signal may be seen with other hepatic malignancies, such as metastases and intrahepatic cholangiocarcinomas [28–30].

Both extracellular and hepatobiliary agents can be used for imaging of the liver. We favour the use of the more expensive hepatobiliary agents only in specific cases where key decisions are to be made with regard to transplant or locoregional treatment. Indeed, hepatobiliary agents can present radiologists with greater diagnostic conundrums in contrast to more clarity.

Extracellular gadolinium-based contrast agents (for example, gadopentetate dimeglumine (Gd-DTPA), Magnevist®, Bayer HealthCare), distribute from the vascular space into the interstitial compartment. The standard dose is 0.1 mmol/kg typically injected intravenously at a rate of 2 mL/s followed by a normal saline “flush” of 20 to 50 mL.

Hepatobiliary agents distribute into the interstitial space, but, importantly for hepatic imaging, are also taken up by hepatocytes with subsequent biliary excretion. Multihance, Bracco Diagnostics, Princeton, NJ, USA) was the first to be approved. Approximately 95% of this agent is excreted by the kidney, but 3 to 5% is taken up by the normal hepatocytes and excreted into the biliary tract. Gadoxetate Disodium (U.S: Eovist, Europe: Primovist, Bayer Healthcare Pharmaceuticals, Wayne, NJ, USA) has approximately 50:50 excretion between renal (glomerular filtration) and hepatocyte uptake/biliary excretion. This can therefore be used for the early dynamic imaging phase in the liver, as above, followed by a 20 min T1-weighted imaging phase where the liver is of higher signal intensity and non-hepatocyte containing masses will be of low signal intensity. Hepatocyte-specific contrast agents have been shown in many studies to increase lesion sensitivity for HCC by capitalizing on evidence that poorly differentiated HCCs do not contain functioning hepatocytes and bile ducts, and therefore demonstrate hypointense signal relative to the surrounding liver parenchyma [30, 31]. Combining contrast-enhanced MRI features and hepatobiliary phase imaging has demonstrated sensitivities and specificities of greater than 90% [31].

Potential pitfalls that apply specifically to Eovist/Primovist include transient marked motion on arterial phase images, inability to assess washout after the portal venous phase due to early parenchymal enhancement, difficulty identifying “capsule appearance” due to hepatic parenchymal enhancement, and difficulty identifying venous tumor invasion due to more rapid venous clearance and decreased vein to liver contrast [32, 33].

The use of hepatobiliary agents for the diagnosis of HCC is in transition. Some major HCC imaging guidelines do not mention this class contrast agents [5, 6, 34], while other societies or organizations recommend their use [35]. It remains unclear whether hepatobiliary phase contrast hypoenhancement [32] will be more widely incorporated in comparison with conventional extracellular contrast agent imaging characteristics for the diagnosis of HCC (Table 1).

## CT technique

Multidetector CT (MDCT) allows fast, high-quality, thin-section imaging and permits 3D reconstruction with better spatial resolution than that of MRI. Fast injection rates (4–8 ml/s) provide more reliable enhancement during the hepatic arterial phase and increase the sensitivity of CT to liver lesions. Studies have demonstrated hypervascular components in 81–89% of HCCs [36]. For patients with contraindications to MRI CT serves as an adequate alternative.

CT imaging technique is based on the same principles as dynamic contrast MRI, using arterial enhancement, delayed washout, and a delayed enhancing pseudocapsule as the pillars of diagnosis. The precontrast images serve as a baseline to gauge subsequent enhancement. Following the injection of 100 ml of Omnipaque 350 (Iohexol) we use a bolus tracking system (threshold attenuation in the aorta 150 HU) to initiate arterial phase breathhold imaging through the liver. Subsequent series of images are taken at 65 s and 240 s to provide venous and delayed phase imaging Subtraction images (postcontrast minus precontrast) may be helpful for detection of enhancement and evaluation of its degree [37] (Table 1).

## Diagnosis of HCC and report writing

**Fig. 1:**
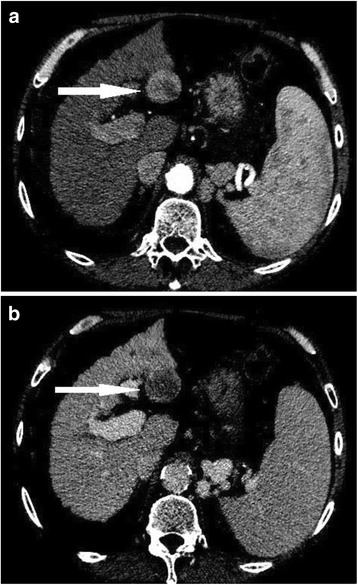
54 year old male with hepatitis C cirrhosis. CT shows an arterial enhancing nodule **a** with washout of contrast in the delayed phase **b** consistent with hepatocellular carcinoma

The hallmark feature of HCC on both CT and MRI is late arterial enhancement with washout relative to the liver parenchyma during the venous or delayed phases (3–5 min post injection) (Fig. 1). This pattern of enhancement has been shown to demonstrate high specificity and positive predictive value [38–40] making it the noninvasive standard for HCC diagnosis [5, 6, 35, 41–44].

**Fig. 2:**
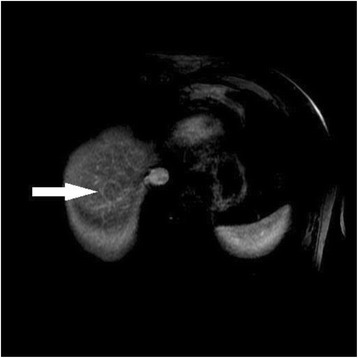
67 year old male with alchohol liver disease and cirrhosis. Venous phase MRI with gadolinium demonstrates an HCC nodule at the dome of the liver with capsular enhancement

In addition to the enhancement pattern, additional features of HCC have been described which are also specific for HCC including capsular enhancement [30, 45, 46]. Capsular enhancement (Fig. 2) is defined as a persistent peripheral enhancing rim seen on venous and delayed phases.

More specific to MRI, a diagnosis of HCC is often attributed to a lesion showing only arterial enhancement or only washout and pseudocapsule formation, if the lesion also demonstrates increased signal intensity on T2-weighted mages [47, 48] or if the lesion restricts diffusion [25, 27, 49], although some caution should be applied to both of these adjuncts as they can result in false positive interpretations [50] (Table 2).

**Table 2:** MRI major and ancillary features for the diagnosis of HCC

HCC: major feature s	HCC: ancillary features
Arterial phase enhancement	T2-w hyperintensity
Delayed phase “washout”	Restriction of diffusion
Threshold growth	Intra-lesional fat
Delayed enhancing capsule	

**Fig. 3:**
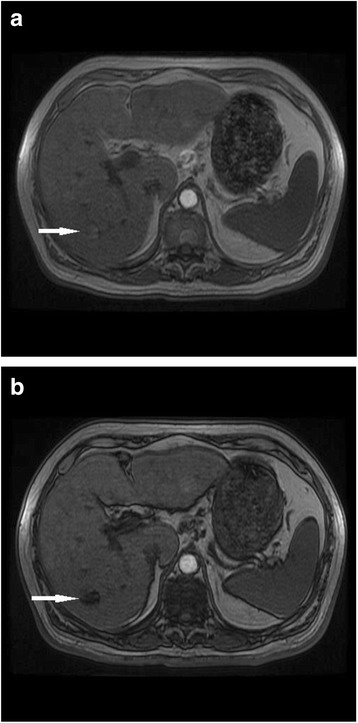
71 year old male with hepatitis C cirrhosis. Signal drop out on opposed phase imaging (**b**) in comparison with in phase imaging (**a**). The findings represent intracellular lipid in an HCC tumour

Intracellular lipid detected within a nodule on dual-echo in and opposed phase T1-weighted MRI is an additional finding which has been shown to be reasonably specific for HCC. This can be a useful addition to the toolbox when looking at a lesion with non-specific enhancement characteristics as intracellular lipid is very rare in a regenerative or dysplastic nodules [51] (Fig. 3).

In the event of uncertainty a consensus opinion is reached from the available liver imaging specialists in the department. Lesions with focal hepatic arterial enhancement, but without washout, capsule enhancement, or abnormal increased T2 signal, are considered dysplastic nodules (if clearly a defined nodule) or nonspecific hypervascular lesions (if nonmarginated and subcapsular).

We review prior imaging and clinical information for all patients. An understanding of the treatment options for HCC under the current guidelines is essential. We structure the conclusions of our reports so that the multidisciplinary liver group can make informed decisions in the context of the options available.

Reports indicate the size (largest axial or coronal section diameter), number, and location of HCC lesions. The Couinaud classification is used for anatomic reference [52]. Although the system was designed for surgical planning it is universally accepted, simple, and more concise than the descriptive terms for the segmental anatomy. The coronal measurement is frequently omitted in reports but is important because it affects the treatment stratification, both for transplant evaluation and for determination of the type of locoregional therapy to be used.

We number the tumors from 1 to 4. If there are more than 4 lesions then we determine whether there is unilobar or bilobar disease and describe how many lesions there are in each lobe, again numbering them so that they can be easily detected. We believe in the importance of providing series and image numbers for each lesion up to 4 lesions so that if the reporting radiologist is not present at the multidisciplinary meeting, or if surgeons or liver specialists are looking at the images, they can find the lesions quickly and not become confused by other confounding imaging findings.

For each lesion the T1-weighted, T2-weighted, diffusion weighted and contrast enhanced characteristics are always described. If there are ancillary findings, for example signal dropout on opposed phase imaging in contrast with in phase imaging, then we add those as well. Although we do not strictly apply a LIRADS (Liver Imaging Reporting and Data System) number to each lesion, we report findings in the context of the LIRADS criteria as these are the current most comprehensive guidelines used to stratify the risk malignancy in the context of cirrhosis and HCC [53]. LIRADS is a useful system to use when there is not close communication in a multidisciplinary setting. It is easily accessible online and the system is helpful for those cases where there is some uncertainty.

For specific examples which are not clearly covered by guidelines, our experiences are that small nodule-like arterial enhancing lesions which do not show associated washout, but which increase in conspicuity over time, merit close attention on follow up imaging as these often develop ancillary features of washout, pseudocapsule or restricted diffusion over time. Small foci of restricted diffusion or high T2-weighted signal with arterial enhancement often turn out to be HCC, whereas small foci or restricted diffusion without arterial enhancement, and without other ancillary features, are very common, and are almost always not related to cancer.

A review for extrahepatic disease is essential as metastatic disease changes all of the treatment pathways. The lungs should be imaged once HCC is diagnosed. Metastatic disease is seen in multiple locations but portal lymph nodes, peritoneum, adrenal glands and bones are the more frequent locations.

## Selection and staging

Once a patient is diagnosed with HCC, a multidisciplinary approach is adopted to determine optimal therapy and further management. Our group includes transplant surgeons, hepatologists, oncologists, radiation oncologists, and cross-sectional and interventional radiologistsWe prepare the cases for presentation each week.

Although several staging schema have been developed, none have been universally adopted. A few main factors have been identified as influential in the prognosis of patients with HCC. These include liver function, tumor size and number, tumor extent, including vascular invasion and extrahepatic spread, evidence of portal hypertension, and clinical performance status. Tumor proximity to large vessels and main bile ducts can also be pertinent with regard to ablative therapies, and is worth mentioning if these treatments are likely to be considered.

CT and MRI are useful in identifying tumor extent and extrahepatic spread. They also provide secondary evidence of portal hypertension, including the presence of splenomegaly and portosystemic collaterals. Imaging of the chest is also recommended as part of the initial work up, given that lung and bone are common sites for HCC metastasis. A bone scan can also be performed if there is a suspicion for osseous metastasis, or if the patient is being considered for liver transplantation.

The Barcelona Clinic Liver Cancer (BCLC) system links the staging of HCC in patients with cirrhosis with treatment options, making it the most commonly adopted staging system [5, 6].

The BCLC system identifies those patients with early stage HCC who may benefit from curative therapies (stage 0 and A), those at intermediate (stage B) or advanced (stage C) stages who may benefit from palliative treatments, and those who are most suitable for best supportive care (stage D). Curative treatment options, including transplantation, resection, and ablation for patients with early stage disease depends on local factors, patient specific issues, and patient preference. Palliative, non-curative treatment options include transcatheter arterial chemoembolization (TACE) for stage B disease, radioembolisation, and sorafenib for advanced stage C disease. TACE is also increasingly used as a “bridge” to transplant, and in some cases to downstage patients so that they can become candidates for a transplant list [54, 55].

In equivocal cases where the diagnosis of HCC is uncertain in small lesions, a reasonable approach is to wait 3 months and image again [56, 57].

## Post therapy imaging

**Fig. 4:**
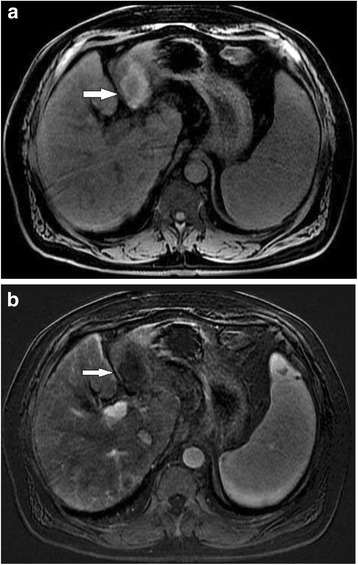
66 year old female with hepatitis C cirrhosis post microwave ablation of HCC Precontrast image post microwave ablation (**a**) show a cavity with intrinsic high signal on T1 weighted imaging. A subtraction image (**b**) removes the high signal resulting in no evidence of enhancement

Because many patients with HCC do not meet criteria for transplantation or surgery, a large proportion of patients receive locoregional therapy or systemic therapy and therefore require post-therapy imaging to evaluate for initial response and recurrent disease. No established guidelines for ideal surveillance time intervals exist. Recurrence is 6.5 times more likely to occur in the first year after therapy than in the second year, so most guidelines suggest 3 monthly interval imaging in the first year after treatment [58]. We follow up with imaging at 3 month intervals for one year followed by 6 month intervals for 2 years, and then we return to ultrasound screening. It is important to use the same modality for each follow up as comparison between CT and MRI can be challenging. We generally use MRI for follow up as the imaging findings can be more difficult to interpret following treatment and the subtraction images can be really useful (Fig. 4).

Several systems have been developed to objectively evaluate the response of HCC to locoregional therapy. Some of these are based on tumor size, such as the WHO (World Health Organisation) and RECIST (Response Evaluation Criteria in Solid Tumours) criteria [59, 60], while others, such as EASL, AASLD, and mRECIST, are based on the assessment of residual enhancing HCC [61, 62]. mRECIST, or modified RECIST, therefore does not evaluate tumour bulk itself, as does RECIST, as this may not change after treatment, or may even increase, but assesses the volume of residual functional tumour or arterial enhancing tissue [63]. Studies have shown that the mRECIST and EASL enhancement-based protocols correlate more accurately with residual disease burden and with survival after therapy than the size-based protocols for patients treated with ablation, radioembolization and TACE [63–67]. At our multidisciplinary meetings we use a combination of mRECIST and EASL criteria to quantify residual or recurrent tumour, along with informed discussion from the team members (Table 3).

**Table 3:** Summary of mRECIST and EASL responses

	mRECIST	EASL
Complete Response	Disappearance of any intratumoral arterial enhancement in all target lesions (up to 2 measurable liver lesions)	Disappearance of any intratumoral arterial enhancement in all measurable arterial enhancing liver lesions
Partial Response	Decrease >30% in the sum of longest diameters of viable target lesions	Decrease >50% in the sum of the product of bidimensional diameters of viable target lesions
Progressive Disease	Increase >20% in the sum of longest diameters of viable target lesions	Increase >25% in the sum of the diameters of viable target lesions
Stable Disease	None of the above	None of the above

**Fig. 5:**
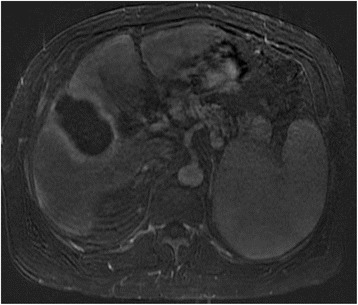
63 year old male with cirrhosis and HCC treated with microwave ablation. A thin rim of enhancement post ablation, consistent with hyperemia adjacent to the ablation zone, is a normal finding and does not represent recurrent tumour

**Fig. 6:**
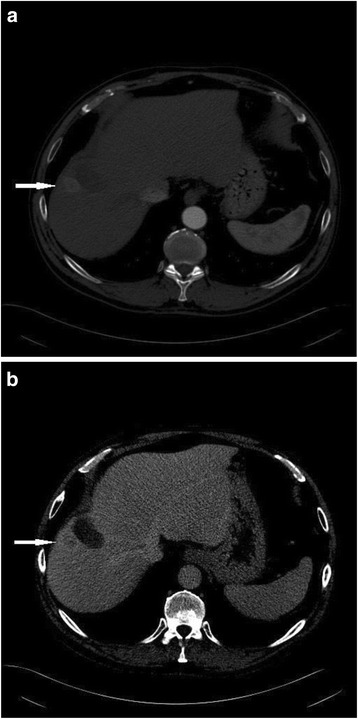
57 year old female with cirrhosis and HCC treated with RFA. CT in arterial (**a**) and venous (**b**) phases shows enhancement and washout of a nodule adjacent to an RFA ablation zone

Prior to reporting we make sure we have established the procedures performed or therapies used, as lack of awareness of these can lead to embarrassing errors in reporting. Regardless of the therapy performed, treated tumor should demonstrate an absence of enhancement. A thin rim of enhancement can be seen as a normal finding after ablation and TACE due to adjacent hyperemia and fibrosis (Fig. 5). However, residual or recurrent disease presents as thick or nodular peripheral arterial enhancement [65, 68, 69] (Fig. 6). Post-ablation changes are similar regardless of what type of ablation is performed. The ablation zone should be larger than the original tumor by between 5 and 10 mm. If it is not, then careful attention to subtle enhancing lesions is needed. Ablation zones can decrease in size with time. An ablation zone can demonstrate high signal intensity on pre-contrast T1-weighted images as a result of coagulative necrosis, making evaluation for arterial enhancement difficult in the absence of subtraction imaging. Subtractions should therefore be routinely included within the MRI protocol [22].

## Conclusions

The accepted modality for hepatocellular carcinoma screening is ultrasound. Once HCC is suspected then CT or MRI may be used to confirm the diagnosis and establish the tumor burden for staging purposes. The BCLC classification system is the most frequently used for treatment planning. However, multidisciplinary meeting and planning is essential to ensure that the correct pathways are adopted within the context of each institution. Following surgical, locoregional, chemotherapeutic or radiotherapeutic treatment, follow up imaging and regular multidisciplinary discussion is adopted.

## Abbreviations

AASLD: American Association for the Study of Liver Diseases
BCLC: Barcelona Clinic Liver Cancer
CT: Computed tomography
DWI: Diffusion weighted imaging
EASL–EORTC: European Association for the Study of the Liver, European Organisation for Research and Treatment of Cancer
FSE: Half-Fourier acquisition turbo spin-echo
Gd: Gadolinium
GRE: Gradient echo
HCC: Hepatocellular carcinoma
LIRADS: Liver Image and Reporting Data System
MDCT: Multidetector computed tomography
mRECIST: modified Response Evaluation Criteria in Solid Tumours
MRI: Magnetic resonance imaging
RECIST: Response Evaluation Criteria in Solid Tumours
TACE: Transcatheter arterial chemoembolization
WHO: World Health Organisation
